# Case Report: Tumor-related hemorrhage determined treatment sequencing in blastic plasmacytoid dendritic cell neoplasm coexisting with gastric cardia adenocarcinoma

**DOI:** 10.3389/fonc.2026.1841250

**Published:** 2026-05-01

**Authors:** Yuze Li, Chenggong Zhang, Xudong Song, Guoquan Tao

**Affiliations:** Department of Gastrointestinal Surgery, The Affiliated Huai’an No. 1 People’s Hospital of Nanjing Medical University, Huai’an, China

**Keywords:** blastic plasmacytoid dendritic cell neoplasm, BPDCN, cardia adenocarcinoma, multiple primary malignancies, upper gastrointestinal bleeding

## Abstract

Blastic plasmacytoid dendritic cell neoplasm (BPDCN) is a rare and highly aggressive hematologic malignancy that commonly presents with skin involvement and may also involve the bone marrow, peripheral blood, and lymph nodes. The coexistence of BPDCN with a primary solid tumor is extremely uncommon and poses substantial diagnostic and therapeutic challenges. We report a 70-year-old man who was first diagnosed with BPDCN after excision of a left forearm lesion and later developed recurrent hematemesis during chemotherapy. Subsequent endoscopic evaluation revealed a bleeding cardia lesion, and repeat biopsy confirmed poorly differentiated gastric cardia adenocarcinoma. After multidisciplinary evaluation, radical gastrectomy was performed first to control tumor-related bleeding, after which systemic chemotherapy for BPDCN was resumed. This case suggests that, in BPDCN patients who develop hematemesis, progressive anemia, or weight loss during treatment, prompt evaluation for a second primary gastrointestinal malignancy is warranted, and control of active tumor-related bleeding may need to precede hematologic therapy.

## 1 Introduction

Blastic plasmacytoid dendritic cell neoplasm (BPDCN) is a rare hematologic malignancy derived from precursors of plasmacytoid dendritic cells. The tumor typically expresses CD4, CD56, and CD123, often together with other plasmacytoid dendritic cell-associated markers and predominantly affects older men with frequent cutaneous involvement at presentation (1–3). Although gastric cancer remains a common malignancy worldwide (4), clearly comparable reports of BPDCN coexisting with a second primary solid tumor remain extremely limited (5, 13). Here, we report a case of BPDCN coexisting with gastric cardia adenocarcinoma in which ongoing tumor-related bleeding determined treatment priority, illustrating how immediate life-threatening complications can outweigh a static oncologic hierarchy in therapeutic sequencing. In this patient, BPDCN was diagnosed first from a cutaneous forearm lesion, whereas the gastric cardia adenocarcinoma was identified later during treatment after recurrent upper gastrointestinal bleeding prompted repeated endoscopic evaluation.

## 2 Case description

### 2.1 Initial presentation

**Figure 1:**
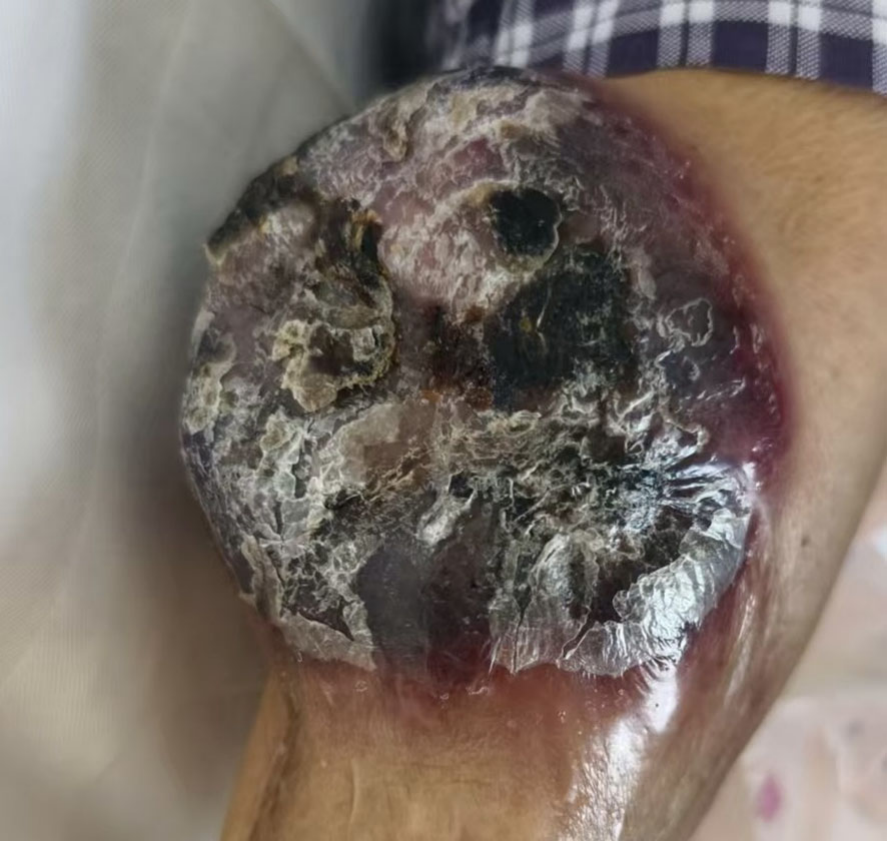
Gross appearance of the left forearm lesion before excision. A dark-red exophytic mass was present near the left elbow, with surface necrosis and crusting.

**Figure 2:**
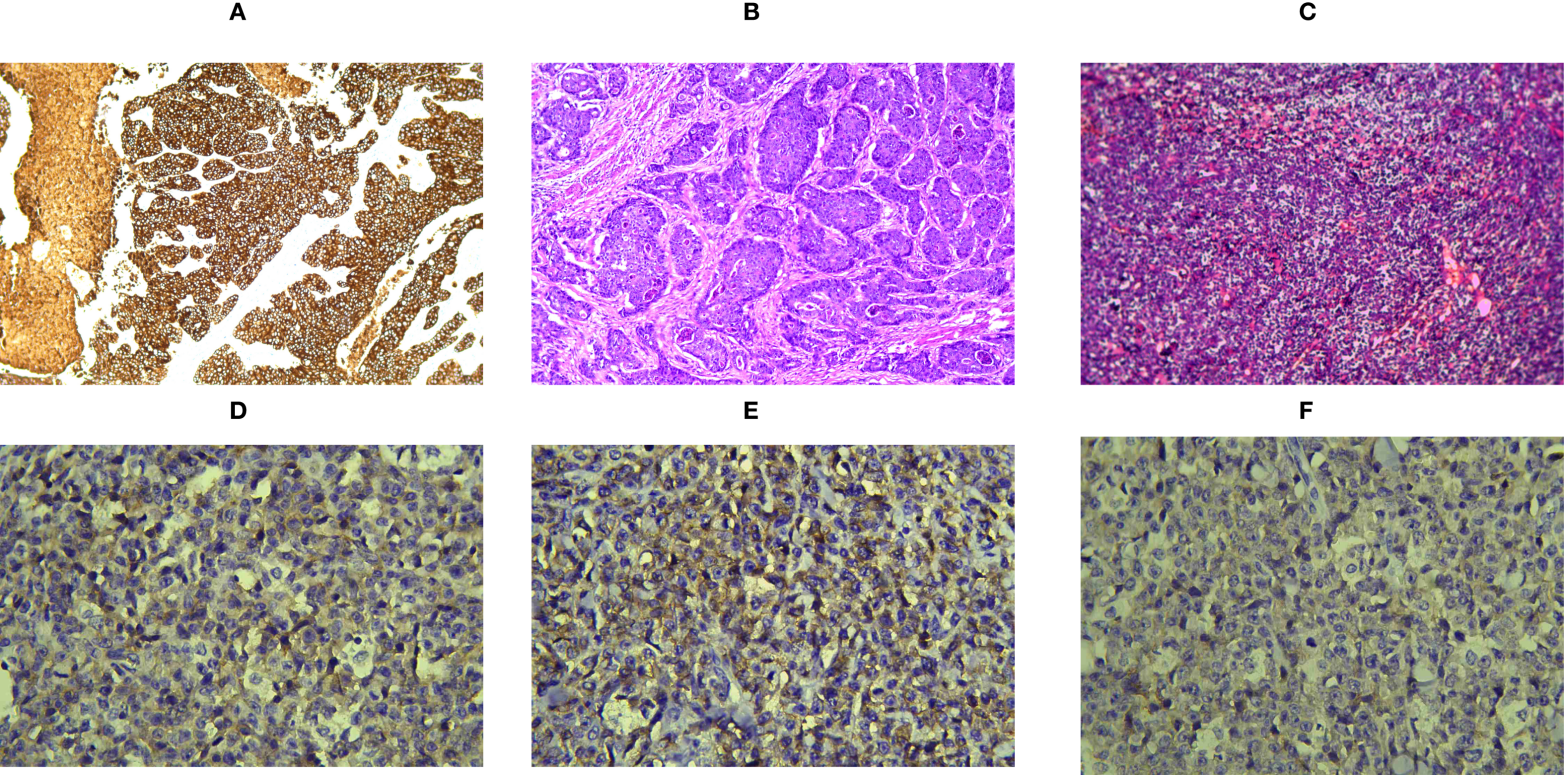
Histopathological findings of the gastric cardia adenocarcinoma and BPDCN in the left forearm lesion. **(A)** Immunohistochemical staining of the cardia tumor showing diffuse CK7 positivity, with epithelial differentiation supported by pan-cytokeratin (AE1/AE3) expression in the immunohistochemical panel. **(B)** Hematoxylin and eosin staining of the cardia tumor showing poorly differentiated adenocarcinoma with infiltrative growth. **(C)** Hematoxylin and eosin staining of the left forearm lesion showing diffuse dermal/subcutaneous infiltration by atypical blastoid cells, representative of BPDCN. **(D)** Immunohistochemical staining of the left forearm lesion showing CD123 positivity in the neoplastic cells. **(E)** Immunohistochemical staining of the left forearm lesion showing CD56 positivity in the neoplastic cells. **(F)** Immunohistochemical staining of the left forearm lesion showing CD4 positivity in the neoplastic cells, further supporting the diagnosis of BPDCN. Total magnification: **(A)** ×100, **(B)** ×100, **(C)** ×100, and **(D–F)** ×400.

For clarity, the patient was first diagnosed with BPDCN in November 2024, whereas the gastric cardia adenocarcinoma was not histologically confirmed until July 2025 after recurrent hematemesis during BPDCN treatment. A 70-year-old man presented to the burn and plastic surgery department of our hospital in November 2024 because of a left forearm mass. Physical examination revealed a firm mass measuring approximately 4 × 5 cm and protruding about 1.5 cm above the skin surface, with a relatively fixed base and a clear border. Excision of the lesion was performed in November 2024. Histopathology showed diffuse proliferation of round cells and short spindle cells. Immunohistochemistry demonstrated CD45/LCA(+), CD4(+), CD33(+), CD43(+), CD56(+), and CD123(+), with a Ki-67 proliferation index of approximately 80%, whereas CD20, PAX5, CD3, CD7, CD8, CD10, CD117, TdT, and MPO were negative. TCF4 and TCL1 immunohistochemical staining were not performed in this case because additional tissue-based testing was not available. Consultation at Fudan University Shanghai Cancer Center supported the pathological diagnosis of BPDCN in the left forearm. However, complete systemic staging was not available because bone marrow examination and peripheral blood/flow cytometry assessment were not performed at initial diagnosis. Postoperative PET-CT performed in December 2024 demonstrated extensive hypermetabolic lesions involving cutaneous and subcutaneous sites, multiple lymph node stations, the paranasal sinuses, and several abdominal and retroperitoneal regions, consistent with multisystem tumor involvement. Collectively, these findings suggested multisystem tumor involvement; however, the relative contribution of BPDCN and the subsequently identified upper gastrointestinal malignancy could not be fully distinguished on imaging alone. A complete BPDCN staging work-up was not available. The gross appearance of the forearm lesion is shown in **Figure 1**, and the representative histopathologic and immunohistochemical images of the BPDCN lesion in the left forearm are provided in **Figures 2C–F**.

### 2.2 Diagnostic assessment

The patient received the first cycle of systemic chemotherapy at another hospital in January 2025 (cyclophosphamide 0.9 g on day 1, liposomal doxorubicin 60 mg on day 1, vincristine 2 mg on day 1, and prednisone 50 mg twice daily on days 1-5). Soon thereafter, he developed hematemesis with severe anemia and was hospitalized twice in the gastroenterology department of our hospital, where his condition improved after supportive treatment such as blood transfusion and fluid replacement. Gastroscopy in January 2025 revealed an actively bleeding mass at the cardia; endoscopic hemostasis was performed, but biopsy could not be obtained because of active bleeding. Because of recurrent bleeding and severe anemia, definitive biopsy could not be obtained during the initial endoscopic evaluation. Repeat gastroscopy performed in July 2025 showed an ulcerated elevated lesion at the cardia, and biopsy confirmed poorly differentiated adenocarcinoma. He was subsequently admitted to our department in August 2025 for definitive management of the cardia lesion. By that time, he had lost approximately 5 kg over the preceding 6 months.

At admission to our surgical department, the temperature was 36.5 °C, heart rate 92 beats/min, respiratory rate 16 breaths/min, and blood pressure 139/81 mmHg. Multiple dark-red nodules and masses of varying sizes were present on the skin, especially on both upper limbs; some were solitary and others confluent, with a firm texture and no obvious tenderness. The abdomen was soft and flat, without tenderness, rebound tenderness, or guarding, and no obvious abdominal mass was palpated.

### 2.3 Therapeutic intervention

Because recurrent hematemesis and severe anemia were considered secondary to bleeding from the cardia lesion, continuation of BPDCN chemotherapy carried a high risk of recurrent hemorrhage. After multidisciplinary discussion, surgical control of the bleeding lesion was prioritized. Later in August 2025, the patient underwent laparoscopic radical proximal gastrectomy with esophagogastrostomy and lymph node dissection. Postoperative pathological examination demonstrated a polypoid poorly differentiated adenocarcinoma at the gastric cardia with invasion into the subserosa, corresponding to a pathological stage of pT3N3 according to the AJCC 8th edition. Immunohistochemical staining showed that the tumor cells were positive for pan-cytokeratin (AE1/AE3) and CK7, weakly positive for Villin and CDX-2, and negative for P40 and CK5/6, supporting the diagnosis of poorly differentiated adenocarcinoma. Additionally, a separate 1.2 cm perigastric nodule adjacent to the lesser curvature was identified. Histopathologic evaluation together with immunohistochemical positivity for CD123 and CD56 confirmed BPDCN involvement in this lesion, in keeping with the patient’s previously confirmed BPDCN. Importantly, the gastric adenocarcinoma itself was not reported to express BPDCN-associated markers; CD123 and CD56 positivity were observed only in the separate perigastric nodule. CD123-targeted therapy, including tagraxofusp, was not administered in this patient because of limited accessibility, the patient’s age, and the immediate need to control ongoing gastrointestinal bleeding.

### 2.4 Follow-up and outcomes

**Figure 3:**
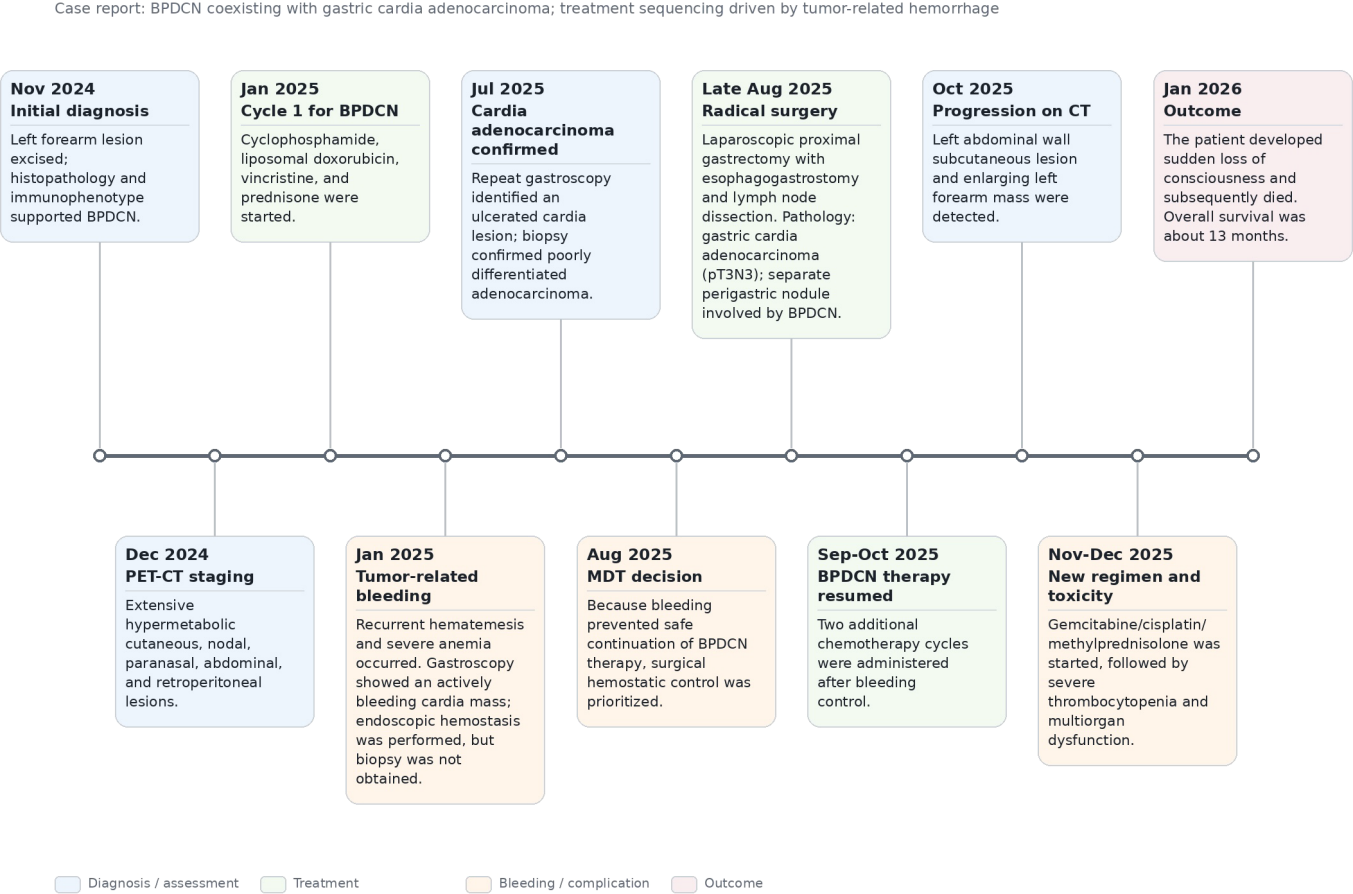
Timeline of diagnosis and treatment in a patient with BPDCN and cardia adenocarcinoma.

In September 2025, the right arm mass enlarged further, and the patient was advised at a cancer hospital to continue chemotherapy. Two additional cycles of chemotherapy were administered in September and October 2025 (cyclophosphamide 0.9 g day 1, liposomal doxorubicin 60 mg day 1, vincristine 2 mg day 1). In October 2025, follow-up CT revealed a subcutaneous metastatic lesion in the left abdominal wall and a large irregular mass on the radial side of the left forearm. The patient then received the first cycle of a new chemotherapy regimen starting in November 2025, consisting of gemcitabine 1.7 g on days 1 and 8, cisplatin 40 mg on days 1–3, and methylprednisolone 500 mg on days 1–3. After chemotherapy, the patient developed severe thrombocytopenia. A second cycle with dose reduction was initiated in December 2025, consisting of gemcitabine 1.4 g on days 1 and 8, cisplatin 32 mg on days 1–3, and methylprednisolone 500 mg on days 1–3. Following the second cycle, the patient developed acid reflux, heartburn, nausea, and vomiting, and was admitted to our hospital. Laboratory tests showed profound thrombocytopenia, accompanied by electrolyte disturbances, cardiac insufficiency, arrhythmia, renal insufficiency, and multiple organ dysfunction. In January 2026, the patient experienced a sudden loss of consciousness. Emergency resuscitation was initiated immediately; however, the family declined further treatment, and the patient was subsequently pronounced dead. The overall survival from initial presentation was approximately 13 months. The terminal clinical course was marked by profound thrombocytopenia, multiorgan dysfunction, and rapid clinical deterioration. The overall diagnostic and therapeutic course is summarized in **Figure 3**.

## 3 Discussion

BPDCN is a rare and aggressive hematologic malignancy that predominantly affects older men and frequently presents with cutaneous lesions (1, 3, 6). Published literature specifically describing BPDCN coexisting with a truly independent second primary solid tumor appears to be extremely limited. Importantly, several published reports describe unusual extramedullary manifestations of BPDCN itself rather than histologically independent synchronous dual primary malignancies. Accordingly, clearly comparable case-level reports remain sparse, although population-level data support the occurrence of second primary malignancies in this setting. In a national database study, 43 of 932 patients with BPDCN (4.61%) developed second primary malignancies, corresponding to an increased risk compared with the general population (SIR 1.43, 95% CI 1.03–1.92) (13). In this context, the present case is distinctive not only because of the coexistence of BPDCN and biopsy-proven gastric cardia adenocarcinoma, but also because active tumor-related bleeding directly determined treatment sequencing and altered the timing of BPDCN-directed therapy.

BPDCN most commonly presents initially with violaceous patches, nodules, or tumor-like cutaneous lesions, and may subsequently be accompanied by bone marrow infiltration–related anemia, thrombocytopenia, and peripheral blood abnormalities, as well as lymphadenopathy and splenomegaly (1, 3, 6). In the present case, the patient initially presented with a mass on the left forearm, followed by the development of multiple cutaneous nodules, which was consistent with the typical skin-first presentation pattern of BPDCN. However, the rapid onset of hematemesis, severe anemia, and weight loss after the first cycle of chemotherapy suggested that these manifestations should not be simply attributed to treatment-related adverse effects or progression of BPDCN itself. In elderly patients with BPDCN, gastrointestinal bleeding, progressive anemia, and unexplained weight loss may reflect not only supportive care–related issues, but also important warning signs of a concomitant primary gastrointestinal malignancy. Early gastroscopy with appropriate histopathologic evaluation is therefore warranted to avoid delaying subsequent therapeutic prioritization and planning.

The diagnosis of BPDCN still relies fundamentally on an integrated assessment of histomorphology and immunophenotype. Classically, the tumor cells express CD4, CD56, and CD123, often together with plasmacytoid dendritic cell–associated markers such as TCL1, CD303, and TCF4, while lacking lineage-specific markers of B-cell, T-cell, and myeloid differentiation (1–3, 7, 8). It should be emphasized that, in a series of 91 cases, only 46% showed concurrent expression of all five characteristic markers—CD4, CD56, CD123, CD303, and TCL1—indicating that BPDCN does not always exhibit a fully typical immunophenotypic profile (3). Therefore, diagnosis should be based on a comprehensive evaluation incorporating morphology, immunohistochemistry, and clinical context (1–3). In recent years, newer immunohistochemical combinations, including TCF4/CD123 dual staining and SOX4/CD123, have further improved the diagnostic reliability of BPDCN. Among these, TCF4/CD123 dual staining showed a detection rate of 100% and a specificity of 99.8% in a cohort of 48 BPDCN cases, while SOX4/CD123 also demonstrated strong diagnostic performance in distinguishing BPDCN from reactive plasmacytoid dendritic cells (7, 8). In the differential diagnosis, BPDCN should be distinguished from acute myeloid leukemia/myeloid sarcoma, acute lymphoblastic leukemia/lymphoma, NK/T-cell lymphoma, and poorly differentiated metastatic carcinoma. In the present case, the left forearm lesion showed a typical immunophenotype of BPDCN, whereas the cardia lesion was pathologically consistent with poorly differentiated adenocarcinoma. The clear differences in morphology and immunophenotypic lineage between the two lesions supported the diagnosis of two primary malignancies rather than dissemination of a single tumor.

With respect to treatment, there is still no fully standardized therapeutic regimen for BPDCN. Traditionally, treatment has largely been adapted from ALL-/AML- or lymphoma-like multi-agent chemotherapy protocols, which can induce initial remission but are associated with high relapse rates and limited durability of response (1, 9). In recent years, CD123-targeted therapy has substantially advanced the treatment landscape of BPDCN (14, 15). In prospective studies, tagraxofusp demonstrated high response rates in previously untreated BPDCN and enabled some patients to proceed to hematopoietic stem cell transplantation as a bridging strategy; it was subsequently approved by the FDA for BPDCN in adults and in children aged 2 years and older (9, 10). In addition, BPDCN appears to be dependent on the BCL2 pathway, and venetoclax-based regimens have provided a new therapeutic option for patients with relapsed/refractory disease or those unable to tolerate intensive treatment (11). For eligible patients, hematopoietic stem cell transplantation remains an important consolidative approach for achieving durable remission, and recent retrospective studies have suggested that allogeneic transplantation may confer superior progression-free and overall survival compared with chemotherapy alone (12). CD123-targeted therapy was not feasible in this patient because of limited accessibility, advanced age, and the need for urgent control of ongoing gastrointestinal bleeding. However, the patient in the present case was elderly and had an ongoing risk of active gastrointestinal bleeding. Accordingly, treatment planning had to prioritize short-term safety and the feasibility of subsequent therapy, rather than mechanically applying the idealized sequence of “standard induction followed by consolidation.”

The most noteworthy aspect of this case was not merely the rarity of the coexistence of BPDCN and cardia carcinoma itself, but rather the determination of treatment sequencing through multidisciplinary discussion. The patient developed hematemesis and severe anemia immediately after the first cycle of BPDCN chemotherapy, indicating that bleeding from the cardia tumor had become the principal obstacle to continuing systemic treatment. Previous studies on gastric cancer–related bleeding have shown that, although endoscopic hemostasis can achieve a relatively high rate of initial bleeding control, short-term rebleeding is not uncommon. In one study including 106 patients with unresectable advanced gastric cancer complicated by bleeding, the 30-day rebleeding rate was 28.3%; another study likewise demonstrated that rebleeding after endoscopic treatment for gastric cancer bleeding was frequent and that early rebleeding was associated with poorer survival (16, 17). Therefore, in selected patients with resectable disease, acceptable performance status, and persistent tumor-related bleeding that precludes further systemic therapy, surgery may serve not only as local treatment for the gastrointestinal malignancy but also as a bridging strategy to restore the feasibility of BPDCN-directed therapy.

In this patient, surgery achieved an important short-term therapeutic goal: no further obvious hematemesis was documented, and systemic therapy for BPDCN could be resumed. However, a single case cannot demonstrate that surgery improves long-term survival. The practical lesson from this case is that when dual primary malignancies coexist and one lesion causes active bleeding that prevents treatment of the other, the most immediately life-threatening problem may need to be addressed first. This report is limited by its single-case nature. BPDCN staging was incomplete because bone marrow examination and peripheral blood/flow cytometric evaluation were not performed at initial diagnosis. In addition to the biopsy-proven gastric cardia adenocarcinoma, a separate perigastric nodule showed histopathologic and immunophenotypic features confirming BPDCN involvement, supporting the coexistence of the two distinct disease processes in this patient. However, the absence of additional BPDCN-associated markers such as TCF4 and TCL1 limited more comprehensive immunophenotypic characterization of the perigastric lesion. Molecular testing, cytogenetic analysis, and next-generation sequencing were not performed on either the BPDCN lesion or the gastric adenocarcinoma; therefore, potential shared or distinct genomic features between the two tumors could not be assessed.

Taken together, this case highlights that, in elderly patients with BPDCN, particularly those who develop hematemesis, melena, progressive anemia, or weight loss during treatment, clinicians should maintain a high index of suspicion for a primary gastrointestinal malignancy and pursue timely diagnostic evaluation. When BPDCN coexists with a solid tumor, therapeutic decision-making should not be based solely on a static hierarchy of oncologic “priority”; instead, treatment should be individualized according to the most immediate life-threatening condition, the feasibility of subsequent systemic therapy, and multidisciplinary comprehensive assessment. This case underscores that, when BPDCN coexists with a bleeding gastrointestinal malignancy, treatment sequencing should be guided by the most immediate life-threatening condition, and timely local control of hemorrhage may be essential for resuming hematologic therapy.

## Data Availability

The original contributions presented in the study are included in the article/supplementary material. Further inquiries can be directed to the corresponding authors.
